# Assessment of the Carcinogenicity of Carbon Nanotubes in the Respiratory System

**DOI:** 10.3390/cancers13061318

**Published:** 2021-03-15

**Authors:** Marcella Barbarino, Antonio Giordano

**Affiliations:** 1 Department of Medical Biotechnologies, University of Siena, 53100 Siena, Italy; antonio.giordano@unisi.it; 2 Sbarro Institute for Cancer Research and Molecular Medicine, Center for Biotechnology, College of Science and Technology, Temple University, Philadelphia, PA 19122, USA

**Keywords:** malignant mesothelioma, carcinogenesis, asbestos exposure, carbon nanotubes

## Abstract

**Simple Summary:**

Malignant mesothelioma is an aggressive cancer of the membranes covering the lung and chest cavity (pleura) or the abdomen (peritoneum), mainly linked to asbestos exposure. Asbestos is a proven human carcinogen but its use is far from being universally banned and the forecasts on the incidence of mesothelioma over the next several years are far from optimistic. Carbon nanotubes are a promising type of nano-materials used in the field of nanotechnology for a wide range of applications. However, the similarities between asbestos and CNTs have raised many concerns about their danger and are still the subject of intense research. Keeping in mind that the asbestos tragedy could have been prevented, the aim of this study is to review the recent scientific evidence on CNTs carcinogenicity.

**Abstract:**

In 2014, the International Agency for Research on Cancer (IARC) classified the first type of carbon nanotubes (CNTs) as possibly carcinogenic to humans, while in the case of other CNTs, it was not possible to ascertain their toxicity due to lack of evidence. Moreover, the physicochemical heterogeneity of this group of substances hamper any generalization on their toxicity. Here, we review the recent relevant toxicity studies produced after the IARC meeting in 2014 on an homogeneous group of CNTs, highlighting the molecular alterations that are relevant for the onset of mesothelioma. Methods: The literature was searched on PubMed and Web of Science for the period 2015–2020, using different combinations keywords. Only data on normal cells of the respiratory system after exposure to fully characterized CNTs for their physico-chemical characteristics were included. Recent studies indicate that CNTs induce a sustained inflammatory response, oxidative stress, fibrosis and histological alterations. The development of mesothelial hyperplasia, mesothelioma, and lungs tumors have been also described in vivo. The data support a strong inflammatory potential of CNTs, similar to that of asbestos, and provide evidence that CNTs exposure led to molecular alterations known to have a key role in mesothelioma onset. These evidences call for an urgent improvement of studies on exposed human populations and adequate systems for monitoring the health of workers exposed to this putative carcinogen.

## 1. Introduction

Malignant pleural mesothelioma (MPM) is an aggressive cancer of the pleural membranes covering the lungs and is strongly linked to asbestos exposure. MPM generally manifests in an advanced stage after a latency period of 30–40 years following asbestos exposure. 

Asbestos is a commercial term describing a group of specific silicate minerals forming bundles of long and thin mineral fibers that, because of their intrinsic characteristic of durability and resistance to chemicals, heat and electricity, were widely used in the late 1800s with the start of the Industrial Revolution. However, as early as 1898, lung damage was described in industry workers exposed to asbestos dust [1] and in the early 1900s, the first reports documenting fibrosis [2,3] and asbestosis [4] in asbestos-exposed workers were published. Only 30 years later (1935), the first association between asbestos and lung cancer was described [5,6] and it was another 10 years passed before asbestos exposure was correlated with pleural tumors, in the work of Wedler in 1943 [7] and the doctorate thesis of Wyres in 1946 [8]. In 1977 [9] and 1987 [10], the International Agency for Research on Cancer (IARC) concluded that asbestos is a human carcinogen and that the size and shape of the fibers influence the incidence of tumors. In 2006 [11] and 2009 [12] asbestos exposure was also correlated with an increased risk of other cancers, such as laryngeal and ovarian cancer.

**Figure 1:**
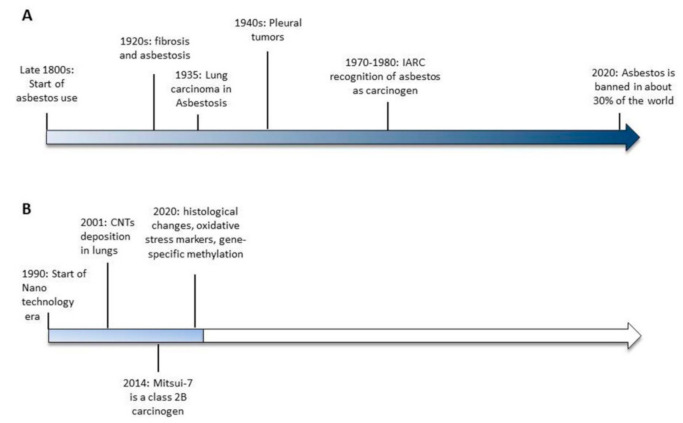
Timelines of the significant events leading to asbestos banning (**A**) and the available evidences of carbon nanotubes-induced toxicity (**B**).

Currently, even though asbestos is a known carcinogen, it is not banned in about 70% of the world (Figure 1). As such, more than 100 years after the recognition of asbestos as a carcinogenic agent, the case is not yet closed (http://ibasecretariat.org/chron_ban_list.php, accessed on 2nd of October 2020). Indeed, it is important to note that countries that banned asbestos a quarter of a century ago are still contributing to the worldwide toll of more than 100,000 asbestos-related deaths per year [13]. As highlighted by Terracini [13], while banning asbestos is important, that alone does not create an asbestos-free environment. It will take a very long time to ban the use of asbestos worldwide, and it will take an even longer time to end up with an environment that is completely safe from the toxic effects of asbestos. For all of these reasons, the forecasts on the incidence of mesothelioma over the next several years are far from optimistic.

It should also be considered that asbestos present in old constructions still represents a daily hazard to human health. There are numerous cases in which the presence of asbestos has been detected during the renovation or demolition of old buildings. The 9/11 terrorist attack in New York City to the World Trade Center, built in the 1970s, created extra exposure of asbestos, the impact of which will be known only in the coming years [14]. In the dense clouds of dust resulting from this tragic event, relevant quantities of carbon nanotubes (CNTs) produced by the high combustion temperatures were also found, along with other pollutants.

CNTs are nanomaterials composed of graphene sheets consisting of a series of carbon rings rolled into cylindrical fibers with an external measurement between 1 and 100 nm. Their fibrous particulate matter, similar to that of asbestos [15] has raised much concern about their safety for human health. In particular, growing evidence supports the idea that inhaled nanomaterials of >5 μm and with a high aspect ratio (3:1), like rod-like carbon nanotubes resembling asbestos, may cause pleural disease including mesothelioma. In 2014, the International Agency for Research on Cancer (IARC) classified the first type of CNT, the long, rigid, needle-shaped Mitsui-7, as possibly carcinogenic to humans (Group 2B) [16], while in the case of other CNTs, it was not possible to ascertain their toxicity due to lack of evidence. It is also important to consider that, together with the lack of sufficient evidence supporting CNTs’ carcinogenicity, their heterogeneity in chemical and physical structures makes it difficult to generalize the available results regarding their possible hazardous effects on human health. 

The present review aims to provide an overview of the recent relevant toxicity studies produced after the IARC meeting in 2014 restricting analysis on a homogeneous group of CNTs: standard materials from the Joint Repository Center (JCR) and well-characterized commercial or in-house-made CNTs produced by catalytic carbon vapor deposition (CVD). Moreover, we review the data on mesothelial and lung cells since the respiratory system is considered the main route of exposure to asbestos and CNTs due to exposure during manufacturing process or to accidental exposure. Therefore, we exclude from our analysis CNTs produced for medical purposes, which are functionalized or modified and, consequently, results obtained from cancer or other models resembling a pathological status. 

This review is structured following the IARC’s parameters [17] for defining an agent as a human carcinogen: induces oxidative stress; induces chronic inflammation; induces epigenetic alterations; is genotoxic; alters DNA repair or causes genomic instability; causes immortalization; alters cell proliferation, cell death, or nutrient supply; acts as an electrophile either directly or after metabolic activation; is immunosuppressive; and modulates receptor-mediated effects. 

## 2. Methods

The literature was searched on PubMed and Web of Science for the period 2015–2020, using different combinations of the following keywords: CNT, carbon nanotubes, SWCNT, MWCNT, single-walled carbon nanotubes, multi-walled carbon nanotubes, genotoxicity, DNA damage, epigenetic, oxidative stress, inflammation, immunosuppression, immortalization, and cytotoxicity. The language was restricted to English. Only data on normal cells of the respiratory system (pleural cells, lung cells, fibroblasts, and lung macrophages) after exposure to reference material (NM-400, NM-401, NM-402, and NM-403), SWCNTs, and MWCNTs synthetized by the CVD method and fully characterized for their physico-chemical characteristics (length, diameter, agglomeration, and surface area) were included in the review.

## 3. An Overview of Carbon Nanotubes

Thirty years ago, the IBM researcher Don Eigler moved the first individual atom using a scanning tunnelling microscope. Despite that progress, Eigler has said he is not sure about when or even if his ideas for computing will bear fruit. It was Eigler who started the era of nanotechnology, the science that is able to create and manipulate materials at the nanoscale. Nano-sized materials, defined as having at least one dimension between 1 and 100 nm, include many types of materials, different in their physicochemical properties, and used in a great variety of applications [18]. Given the immense potential of nanotechnology, the global nanotechnology market has been estimated to reach 126.8 billion U.S. dollars by 2027 [19]. 

The big world of nanotechnology comprises various types of nanomaterial, all differing in their chemo-physical properties. CNTs are the most promising type of nanomaterials in the industry today. They are defined as nanotubes composed of carbon, consisting of one or more cylindrical graphene layers and are classified, on the basis of the number of graphene layers, as single- or multi-walled carbon nanotubes (respectively, SWCNTs and MWCNTs). Larger MWCNTs can contain hundreds of concentric layers.

As CNTs come to be used in a wider range of products, human exposure can take place through various routes, such as local (in medical applications, such as drug delivery, cancer therapy, medical diagnostics and imaging), environmental (industrial waste or accidentally released by the final product), or pulmonary (during occupational handling or accidental exposure). The work environment is actually thought to be the principal source of human exposure to CNTs during the phases of their production, as seen for example in laboratory handling and packaging of the final product, and in this case the most plausible route of exposure to manufactured nanomaterials remains pulmonary inhalation. The inhalation of particles during their synthesis is a significant concern in the growing nanotechnology field. 

Despite different governmental organizations monitoring CNT exposure in workers, there are still no standards for defining the risk levels for CNT exposure. The method of monitoring CNTs in work environments involves measurement of Elemental Carbon (EC). The National Institute for Occupational Safety and Health (NIOSH, USA), based on quantification limits and not on studies in exposed workers, recommends an exposure level of 1 μg/m^3^ elemental carbon (EC) [20]. This limit, which might not be representative of a safe exposure limit, has often been found to be much lower than those measured in various industries, ranging from 2.6 µg/m^3^ to 45 µg/m^3^ depending on the particular workplace analyzed (handling facilities, production areas, construction sites, offices, etc.) [21,22].

The pulmonary toxicity of fibrous materials such as asbestos has been demonstrated to result from deposition (thin fibers deposit in the lungs more efficiently than thick fibers) and tissue persistence (“biopersistence” is directly related to fiber length and inversely related to dissolution and fragmentation rates). CNTs have been demonstrated to deposit in human lungs and other organs. Lung biopsies of people exposed to the dense clouds of dust during the tragic events of 9/11 in New York City have shown the presence of CNTs produced by high combustion temperatures. The first adverse health effects diagnosed were pulmonary fibrosis, and bronchiolocentric parenchymal and granulomatous diseases [14].

Carbon nanotubes, although a sub-group in the immense word of nanomaterials, comprise various substances that differ from each other in length, size, diameter, impurities, and method used for synthesis and dispersion of the final product, among other characteristics. All of these characteristics impact their biological effects, and it is now recognized that generalized conclusions about CNTs should not be drawn by extrapolating data that are available on similar, but not identical, compounds. For these reasons, we focused our analysis on the results obtained using reference CNTs (NM-400, NM-401, NM-402, and NM-403) (https://publications.jrc.ec.europa.eu/repository/bitstream/JRC91205/mwcnt-online.pdf accessed on 15th October 2020), with fully characterized commercial and in-house CNTs produced using the CVD method, which is currently one of the principal techniques used for CNT synthesis. Data regarding CNTs that had been chemically modified to alter their properties and data obtained in cancer cells were excluded from our analysis; this model is suitable for other purposes, such as drug-delivery studies, which are not the focus of this review. 

We reported data relevant to assessing the potential adverse respiratory effects following the IARC’s protocol for defining an agent as a human carcinogen [17]. For each group of characteristics, we analyzed data obtained from in vitro models of pleura, lung macrophages, and airway cells, from in vivo studies examining effects on the respiratory system, and from biological fluids collected from exposed workers, highlighting those results that could be relevant for mesothelioma onset.

## 4. Carbon Nanotubes and the Hallmarks of Cancer

### 4.1. Oxidative Stress, Chronic Inflammation 

The oxidative potential of a particle is the intrinsic property to form reactive oxygen species (ROS). Generation of ROS and free radicals has been demonstrated to be involved in the molecular mechanisms leading to mesothelioma as well as other asbestos-related diseases. In cell-free systems, asbestos can generate free radicals and induce release of inflammatory mediators such as cytokines, growth factors, reactive oxygen and nitrogen species in neutrophils, and alveolar macrophages for incomplete/frustrated phagocytosis of fibers. At cellular level, in asbestos exposed cells, inflammation, oxidative stress, and carcinogenesis has been associated with the alteration of the iron metabolism due to iron accumulation on fibers [23]. Similarly, iron impurities in CNTs have been demonstrated to participate in increased inflammation and oxidative stress in CNTs exposed mesothelial cells, in a “dose-dependent” manner [24,25,26]. 

At the molecular level, ROS may cause different injuries, such as gene mutations and structural alterations to the DNA, leading to deregulation in cell proliferation and apoptosis. Oxidative DNA damage is often characteristic of chronic inflammation, one of the main mechanisms underlying mesothelial transformation.

During the inflammation process, the cross-talk between inflammatory cells and damaged alveolar cells has been recognized to contribute to mesothelioma pathogenesis as well as other respiratory disease like lung fibrosis and lung cancer [27,28]. Lung fibrosis manifests with excessive deposition of collagen fibers in the extracellular matrix (ECM) and remodelling of the alveolar parenchyma, leading to a progressive loss of lung function. It includes a first acute inflammation phase where inflammatory cells infiltrate the tissue, secrete proinflammatory mediators (cytokines TNFα, IL1α, IL1β, IL6, chemokine CCL2, and fibrogenic growth factors TGF-β1 and PDGF-A), and collagen is deposited in the ECM. After this early response, granulomatous fibrotic foci deposits around the lesions are detectable. Activation of fibroblasts and formation of myofibroblasts (fibroblast-to-myofibroblast transition) and epithelial-to-mesenchymal transition (EMT) of alveolar type II cells are drivers of this process [29,30]. Lung fibrosis is one of the first documented injuries to lung described in asbestos-exposed workers 2,3 and the inflammatory process leading to fibrosis has been well characterized using long, needle-like Mitsui-7 MWCNT exposure in vivo [31,32]. The role of oxidative stress in CNTs-induced lung fibrosis was demonstrated through the use of the antioxidant N-Acetyl Cysteine, which interfered with NLRP3 inflammasome activation and generation of pulmonary fibrosis in mice [33].

In both asbestos- and MWCNT-exposed workers, markers of fibrosis, profibrotic inflammatory mediators and immune markers [21,34,35], as well as dysregulation in mRNAs and target genes linked to the activation of key pathways involved in several disease outcomes (e.g., cancer, respiratory and cardiovascular disease, and fibrosis) [36] have been found. Markers of oxidative stress and mitochondrial dysfunction have also been found in exposed workers [37]. 

The similarity between MWCNTs and asbestos due to their inflammatory and oxidative potential has been recently demonstrated in vivo with long MWCNTs (Nanostructured & Amorphous Materials, Houston, TX, USA; University of Manchester, Manchester, UK) and long fiber amosite asbestos instilled into the pleural cavity of mice. Exposure to long fibers but not to short fibers resulted in the development and progression of inflammatory lesions along the pleura and in the increase of markers of oxidative stress and genotoxicity. All exposed animals displayed pleural lesions (mesothelial hyperplasia and fibrosis), and chronic inflammation and, in 10–25% of animals exposed to long MWCNTs, the lesions progressed to pleural mesothelioma [38]. Different results were obtained with long NM-401 and Mitsui-7 MWCNTs. In this study, toxicity and inflammation were observed only in mice exposed to short MWCNTs (NM-400, NM-402, NM-403, and MWCNTs from CheapTubes, Brattleboro, VT, USA) [39].

However, other studies in vivo have demonstrated that both long and short industrial MWCNTs induced granulomatous changes in the lungs, development of pulmonary fibrosis, and inflammation accompanied by increase in vimentin, TGF-beta, IL-1b, IL-18, and cardiac fibrotic deposition [40,41,42,43,44]. Commercial short MWCNTs (tangled) (Graphistrength© C100; Arkema, France) showed prolonged TNF-α release in BAL of exposed rats associated with increased collagen staining [45]. 

Similar results were obtained with SWCNTs (Nikkiso Co., Ltd., Tokyo, Japan), showing strong persistent pulmonary inflammation [46]. The same group also demonstrated that the shorter the length of SWCNTs is, the stronger the toxicity. Short SWCNTs (Nikkiso Co., LTD., Tokyo, Japan) with a length of 2.8 µm induced a weaker inflammatory response and pulmonary toxicity than those with a length of 0.4 µm [42].

It has also been demonstrated that chronic exposure to commercial short SWCNTs (CNI, Houston, TX, USA) induces tumor growth (subcutaneously injected) and metastasis to liver and lung through activation of EMT [47]. Cancer development (Bronchiolo-alveolar adenoma and carcinoma) was also found in 18% of mice exposed to a single intratracheal instillation of short SWCNT (Nikkiso Co., Ltd., Tokyo, Japan) [46].

For a long time, length has been considered a predictor of CNTs’ adverse biological effects. However, even if this is true in some cases, many in vitro studies support the concept that the length of CNTs might not be a unique determinant of the biological response. Recently, shape and diameter have been correlated with accessibility to the macrophage interior subsequently affecting their degradation ability and, therefore, ROS production. Since alveolar clearance contributes to inhalation toxicity, the understanding of parameters predicting CNT toxicity is of crucial importance. This question has been challenged in many studies. Rigid, needle-shaped, long Mitsui-7 MWCNTs (diameter > 50 µm), which are poorly uptaken into phagosomes of alveolar macrophages, have been demonstrated to not induce ROS release. On the contrary, curved, straight, long and thin MWCNTs from different manufacturers, with diameters <20 µm which localize in vacuole-like compartments, have been demonstrated to generate intracellular ROS. For all the analyzed MWCNTs, increased levels of pro-inflammatory cytokines (IL-1α, IL-1β, MIP-1α, INF-γ, IL-18, MCP-1, and TNF-α) were found, implying that the inflammatory response might not be strictly related to the phagocytic ability of the macrophages [48]. ROS production from lung cells could be responsible for the inflammatory response of macrophages in the absence of phagocytic activity. While the rigid, straight, “needle-like” NM-401 MWCNTs, which are similar to Mitsui-7, are poorly uptaken by macrophages and do not cause an increase in NO production, lung fibroblast cells (V79) were demonstrated to be able to uptake NM-401, with 80% of fibers localized in endosomes, generating a consistent production of intracellular ROS [49]. Short NM-400 and NM-402 MWCNTs with a diameter <20 µm, are instead efficiently degraded by macrophages and induce an increase in NO accompanied by acute inflammation [50]. 

Markers of inflammation and oxidative stress were also studied in epithelial cells. Induction of oxidative stress have been described in lung epithelial cells exposed to NM-402 and NM-403 with values comparable to or higher than that of Mitsui-7 [51] while in BEAS-2B cells, a significant reduction in the levels of mRNA expression of pro-inflammatory cytokines IL-1β, IL-6 and IL-8 and an increase in the antioxidant HO-1 gene were found in long-term exposure (three weeks) to NM-403 [52]. However the authors associated these contradictory findings to the metal contaminants present in NM-403.

As a driver of lung fibrosis, the activation of the EMT program in lung epithelial cells by fibrous materials has been documented in four different studies in airway epithelial cells. Exposure to chrysotile asbestos, SWCNTs, Mitsui-7, and Mitsui-7-derived MWCNTs with the length reduced to 1.12 µm, at sub-toxic concentrations led to an increase in mesenchymal markers (α-smooth muscle actin, vimentin, metalloproteinases, and fibronectin), a decrease in epithelial markers (E-cadherin and β-catenin), and activation of the TGF-β–mediated signaling pathway [40,53,54]. 

Fibrogenic potential was also demonstrated with an in-house lung microtissue array device in airway epithelial cells exposed to non-toxic concentrations of short MWCNTs (CheapTubes.com accessed on 15th of October 2020) together with a significant increase in expression of the fibrogenic marker miR-21. These effects were not found in cells exposed to long MWCNTs [55]. 

All of the results reported above indicate that physico-chemical characteristics such as length and diameter could partially explain the different biological responses but, alone, might not be predictive of inflammatory response. Many variables such as the presence of CNTs of different lengths in the same preparation together with their heterogeneity in experimental settings contribute to the difficulty in predicting their inflammatory and oxidative effects. Particularly in in vivo studies (Table 1 and Table 2), different route of exposure and different endpoints analyzed have been used for the evaluation of pathological parameters. Even though studies comparing the inhalation and instillation of MWCNT showed that both methods induced pulmonary inflammation [56], inhalation is more powerful in inducing inflammation [57] and should be the preferred method for studies on accidental exposure during the manufacturing process since it recreates real situations better. 

### 4.2. Epigenetic Alterations

It is well known that epigenetic changes in DNA and RNA play an important role in the regulation of gene expression by changing DNA accessibility to the cellular machinery, and switching on/off gene expression. As indicators of environmental insults, the study of epigenetics is a useful tool to understand disease-related mechanisms as well as serve as an indicator of disease risk. Among the epigenetic modifications affecting the genome, DNA methylation, the process by which a methyl group is added to carbon five in the cytosine pyridine ring forming 5-methylcytosine (5 mC) in DNA, is the most studied for the assessment of the potential hazard of fiber-like materials. Mesothelioma, as well other asbestos-related diseases, has been related to epigenetic changes, and the methylation changes of blood markers have been proposed as diagnostic and prognostic markers for mesothelioma [61,62,63]. In recent epidemiological studies in asbestos-exposed populations, a decrease in the levels of blood global 5-methylcytosine (5 mC) has been described in both healthy exposed workers and in those with benign asbestos-related disorders, confirming that global methylation could be a useful marker of asbestos exposure but, unfortunately, cannot be used as indicator of asbestos-related disease [64,65].

In MWCNT-exposed workers, changes in the methylation of specific genes mainly involved in DNA damage repair, cell cycle regulation, chromatin remodelling, and transcriptional repression (DNMT1, ATM, SKI and HDAC4 promoter) was described in a cross-sectional study [22]. Unlike with asbestos, no significant difference was found in total DNA methylation.

Hypermethylation of specific genes was also found in mice exposed to long MWCNTs (Nanostructured & Amorphous Materials, Houston, TX, USA; University of Manchester, UK) and long amosite fibers, which caused chronic inflammatory lesions or mesothelioma. Of particular importance is the epigenetic silencing of the *CDKN2A* locus, a well-known driver mutation in asbestos-induced mesothelioma, observed in mice exposed to both long MWCNTs and long amosite fibers [38].

Many in vitro studies have confirmed the methylation of specific genes. In 16HBE airway epithelial cells, in-house synthesized short MWCNTs and SWCNTs induced differentially methylated and expressed genes in cellular pathways related to DNA damage repair and cell cycle, with more pronounced effects in MWCNTs. No alteration of global DNA methylation was found [66]. An increased alteration on CpG sites after short -and long-term exposure has also been described for both benchmark short NM-400 MWCNTs and asbestos (CDKN1A and ATM among others) [66,67,68,69,70].

Together with specific gene methylation, other studies have also found a strong genome-wide DNA hypomethylation in airway epithelial cells (BEAS-2B and 16HBE) exposed to commercial short MWCNTs (CheapTubes, Brattleboro, VT, USA) and NM-400 and NM-401 [67,68,69,71].

It is important to note that most of the hypomethylated genes observed after two weeks of exposure to NM-401 became hypermethylated after four weeks of exposure [67], thus highlighting how time and particle type can trigger different and apparently discordant results. 

In conclusion, many studies have demonstrated that change in methylation can be used as a marker of exposure to CNTs but heterogeneity of this class of nanomaterial does not allow for making generalizations. More studies are needed to expand our knowledge about epigenetic regulation of specific genes after CNT exposure. Given our current knowledge of asbestos, we know what genes are strictly linked to mesothelioma onset, and the results regarding epigenetic changes reported above suggest that CNTs could act via a similar mechanism.

### 4.3. Genotoxicity, Alteration in DNA Repair, and Genome Instability

Genotoxic effects can result from primary or secondary mechanisms. The first implies a direct interaction with the genetic material, the latter the oxidation of DNA by reactive oxygen/nitrogen species (ROS/RNS) generated during substance-induced inflammation. Both mechanisms could be involved in the genotoxic response elicited by MWCNTs. 

Although CNTs are considered by IARC to be usually non-reactive and, for Mitsui-7 genotoxicity, have been demonstrated to act via secondary mechanisms, it cannot be excluded that defects in their structure occurring during the synthesis or functionalization could increase their reactivity [72,73]. Very recently, for long and short SWCNTs, the nucleus has been hypothesized to be the primary target site with DNA damage likely due to mechanical penetration [74].

Many studies, such as those described above, support the hypothesis that CNT genotoxicity could result from secondary mechanisms triggered by a strong inflammatory response and ROS release.

A genotoxicity study recently conducted in workers exposed to CNTs (unspecified manufacturer), revealed an 18.3% increase in telomere length and a 35.2% increase in mitochondrial DNA copy number from peripheral blood [75]. 

Asbestos-induced mesothelioma has been linked to polyploidization and aneuploidization, and MWCNTs seem to have similar adverse effects [76]. Chromosomal aberrations (polyploidy), and mitotic and chromosomal disruptions have been demonstrated for commercial MWCNTs (Hodogaya Chemical, Tokyo, Japan; Tokyo Chemical Industry, Tokyo, Japan; Showa Denko K.K, Tokyo, Japan), including MWCNT-7, with different length and shape (including straight fibrous, not straight fibrous (curved), and tangled MWCNTs) in Chinese hamster lung cell lines with straight fibrous being the more potent inducers of polyploidy. None of the seven MWCNTs analyzed caused structural chromosomal aberrations [76]. In the same model, NM-401 was found to be genotoxic, increasing HPRT mutant frequency [49].

In vivo experiments with long MWCNTs (Mitsui & Co. Ltd., Tokyo, Japan) showed a significant increase in DNA damage (comet assay) in the cells of lungs with straight MWCNTs but not with tangled MWCNTs. Moreover, straight MWCNTs caused an increase in DNA strand breaks in BAL cells collected after inhalation but not after pharyngeal aspiration [77]. DNA strand breaks were also observed after intratracheal instillation of straight NM-401 MWCNTs in the transgenic Muta^TM^Mouse model. Moreover, both straight NM-401 and Mitsui-7 MWCNTs increased p53 expression predominantly in the area of fibrotic lesions (more pronounced for NM-401), and induced chronic inflammation and changes in the expression of genes linked to hallmarks of cancer. There was no evidence of a LacZ mutation [58].

Short commercial MWCNTs comprised of straight and tangled MWCNTs (CheapTube, Brattleboro, VT, USA), were demonstrated to induce a dose- and time- dependent neutrophil influx in BAL and to cause DNA damage in the lungs of mice exposed by intratracheal instillation, with large MWCNTs diameter associated with increased genotoxicity (Analysis at 1, 28 and 92 days after exposure). All MWCNTs analyzed induced similar histological changes [60]. 

Another study using commercial short tangled MWCNTs (Graphistrength© C100; Arkema, France) did not disclose genotoxicity in lung cells or a microscopic change in the pleura. As the authors hypothesized, these effects could in part be ascribed to the formation of agglomerates that are poorly uptaken by cells [45]. However, the lack of a positive control in the experimental setting could represent a weakness in the study.

Similar results have been seen in in vitro studies. Long-term exposure of primary human airway epithelial cells (SAECs) to commercial short SWCNTs (CNI, Houston, TX, USA), long Mitsui-7 MWCNTs (Mitsui & Co., Ltd., Tokyo, Japan) and Crocidolite, and mesothelial MeT-5A cells exposed to commercial long MWCNTs (Sigma-Aldrich, St Louis, MO, USA) have demonstrated a substantial increase in DNA damage in γH2A.X foci and p53 dysregulation [54,78]. 

Chromosome damage and chromosome mis-segregation have also been described in airway epithelial cells chronically exposed to sub-toxic doses of short NM-400 and NM-403 MWCNTs [52], while no primary DNA damage or oxidized DNA bases have been observed in short-term experiments with NM-400, NM-401, and NM-403 [50,79,80]

Contrasting results for Micronuclei (MN) formation assay were found in NM-401-exposed cells, according to the different methods used. With the cytokinesis-blocked micronucleus assay (CBMN), authors did not observe significant increases in the frequency of micronucleated binucleated cells or induction of DNA damage by the comet assay [81]. When analyzed by flow cytometry, NM-401 at 20 and 50 µg/mL were able to increase the MN formation [79]. No genotoxic effects with the CBMN assay were detected also for NM-400, NM-402, and NM-403 [81].

Bacterial reverse mutation tests and chromosomal aberration tests, according to the Organization for Economic Co-operation and Development (OECD) Guidelines for Testing of Chemicals, were conducted on straight, long, thin MWCNTs, revealing no structural or numerical chromosomal aberrations below a concentration of 50 µg/mL following short-term exposure, both with and without metabolic activation [48]. However, this test is not suitable for studies with nanomaterials since they are not able to enter the bacterial cell wall, thus leading to the production of false-negative results.

Even though a definitive conclusion on the genotoxicity of CNTs is still impossible to draw, many results have indicated the presence of damaged DNA after exposure to CNTs. It is clear that for genotoxicity assessment, many variables, in addition to those mentioned previously, could interfere with the results. In particular, due to different responses in terms of DNA repair of different cell types, in vitro and in vivo models used represent a key factor together with the dose and time chosen for the analysis. 

### 4.4. Immortalization, Altered Cell Proliferation, Cell Death, or Nutrient Supply

MWCNT-7 carcinogenicity has been demonstrated by different studies in mice in which the whole body has been exposed [82,83]. Nikkiso MWCNTs, which is similar to Mitsui-7, have also been demonstrated to induce pleural malignant mesothelioma and lung tumors in intratracheal instillation studies [59].

The transformation potential in vitro has been documented in different studies. After long-term exposure to commercial long MWCNTs (Sigma-Aldrich, St Louis, MO, USA), mesothelial MeT-5A cells showed features resembling a malignant transformation process and specifically an increase in cell proliferation and invasion capacity, morphology change, and DNA damage [78]. 

Similarly, after long-term exposure to short SWCNTs (CNI, Houston, TX, USA), Mitsui-7 (Mitsui & Co., Ltd., Tokyo, Japan) and Crocidolite asbestos, primary human small airway epithelial cells (SAECs) exhibited neoplastic and cancer stem cell-like properties, such as anchorage-independent colony formation, spheroid formation, anoikis resistance, and expression of cancer stem cell markers [54].

Altered cell proliferation was also described. Cell growth inhibition with benchmark NM-403 MWCNTs [52], and NM-400 and NM401 MWCNTs have been demonstrated in bronchial epithelial cells in long-term experiments [84] and, for NM401 and NM403, in short-term experiments without significant cytotoxicity [51]. 

Similar results were obtained with commercial short SWCNTs and MWCNTs (SES Research, USA; Heji, Hong Kong, China), in lung fibroblasts and in epithelial cells with short rod-like SWCNTs and straight MWCNTs showing higher toxicity [77,85,86]. 

Toxicity studies in macrophages mostly supported the hypothesis that rigidity and high diameters are as key factors underlying toxicity. Indeed, exposure to rigid, needle-shaped Mitsui-7 MWCNTs, and Nikkiso and NM-401 MWCNTs all induce cytotoxicity in macrophage cells while NM-400 and NM-402 did not [48,50]. However, the opposite has also been described in rat alveolar macrophages acutely exposed to highly bent, low-diameter NM-403 MWCNTs, which induced significant toxicity [87].

### 4.5. Immunosuppression, Modulation of Receptor-Mediated Effects, and Electrophilicity

Few data are available regarding the characteristics grouped below. 

Available studies have demonstrated that CNTs can interact and activate the complement system, a key part of the immune system, and induce an early and sustained immunosuppressive response [44,88,89,90]. Moreover, it has been shown that SWCNT exposure in mice increases susceptibility to respiratory viral infections [91].

The ability of CNTs to act as an electrophile and then interact with cellular macromolecules, such as DNA, RNA, lipids, and proteins, has not been thoroughly investigated. It has been suggested that SWCNTs block K+ channel subunits by “plugging” the channel by virtue of the small diameter [92] and interact with TLR4 by hydrophobic interactions [93]. 

All of these studies suggest that the immunosuppression and modulation of the immune responses elicited by CNTs need further investigation. Indeed, an increased susceptibility to pathogens as well as immunosuppression could be a new and potentially significant mechanism of toxicity in humans.

## 5. Discussion

Nanotechnology is changing our world and is believed that it will improve our lives in the near future. CNTs are indeed remarkably valuable given their applications, ranging from drug delivery to electronics. Since we are at the beginning of the nanotechnology era, elucidation of the putative carcinogenicity of CNTs is also at the beginning. Intensive research is underway to understand their safety for human health and a remarkable data pool is being produced using different types of CNTs, models, methods, duration of exposure, amount of CNTs, and time points analyzed. While such heterogeneity is yielding many important results, it is, on the other hand, complicating the evaluation of the danger of CNTs. This situation well reflects the heterogeneity of this class of compounds as well as the different applications intended for their use, thereby making it particularly challenging to identify common features predicting their toxicity. It is not yet understood which aspects of carbon nanomaterials, e.g., surface areas, mass concentrations, lengths or a combination of these features or other factors, influence their toxicity. In addition, establishing criteria for preparation and dispersion, concentrations, models and methods to use, and also including reference materials, will undoubtedly play a crucial role in determining the reliability, reproducibility and comparability of data. In recent years, great improvements have been made in this direction and most non-human-based studies have reported a detailed description of the physiochemical characteristics of CNTs, the method used for their synthesis, the dispersion protocol and the percentage of the impurities present. However, despite these efforts, the lack of a complete characterization of CNT exposure in workers remains a crucial consideration. The type of CNTs varies both across companies and within them over time. Furthermore, in epidemiological studies, there is a high variability among instruments used for sampling and analysis of exposure, and there is still a low number of participants. All of these weaknesses, together with the lack of specific legislation addressing manufacturing processes for nanomaterials, make a direct comparison between studies difficult. 

**Figure 2:**
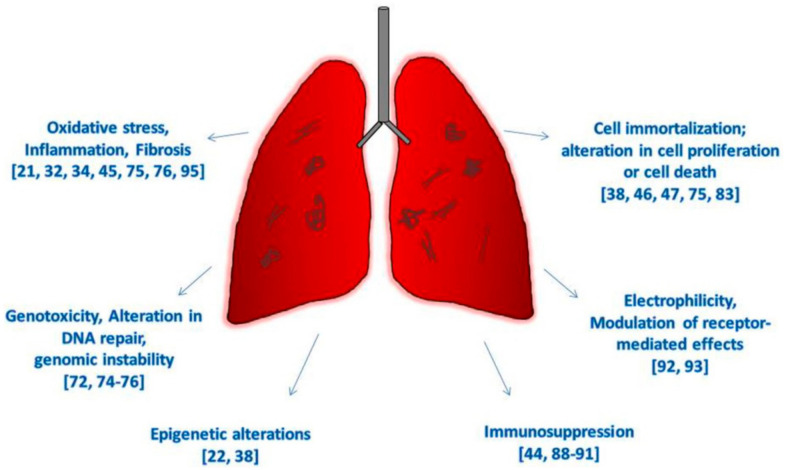
Hallmarks of cancer due to CNTs exposure in vivo and on human-based studies.

However, since the last IARC evaluation of CNT carcinogenicity, conducted in 2014, when enough evidence was available only for Mitsui-7, nine new studies have been performed on humans exposed to CNTs in the workplace, documenting markers of fibrosis, profibrotic inflammatory mediators, and immune markers [21,34,35,94]; epigenetic changes in genes related to DNA repair, cell cycle and repression of transcription [22]; deregulation in pathways and signaling networks linked to pulmonary and carcinogenic outcomes [36]; increase of oxidative markers in the exhaled breath condensates [37], increase in mtDNA copy number [75]; and development of respiratory allergies [95]. Recent findings in vivo have clearly indicated that CNTs induce a sustained inflammatory response and oxidative stress, and fibrosis and histological alterations have been documented in animals exposed to MWCNTs (Table 1) and SWCNTs (Table 2) by inhalation, aspiration, and tracheal instillation [32,44,58]. The development of mesothelial hyperplasia, mesothelioma, and lung tumors have been also described with SWCNTs and long fibers of both asbestos and MWCNTs [32,38,46,59] (Figure 2).

Less evidence is available for assessing cytotoxicity and genotoxicity and we are still far from reaching a consensus. It is nevertheless important to note that there are, however, new findings indicating DNA damage and gene-specific methylations after CNT exposure. In particular, the epigenetic silencing of the *CDKN2A* locus, a well-known driver mutation in asbestos-induced mesothelioma, has been documented in mice exposed to commercial long MWCNTs together with the loss of p16 and p19 protein expression [38]. 

In light of these recent studies analyzed, we agree with the need to evoke a global improvement of studies on exposed human populations as well as with the non-applicability of disproportionate precautionary measures of exposure control. However, considering the absence of any global agreement about the hazards of CNTs, we cannot take the risk creating another man-made tragedy like the case of asbestos where a century passed before its carcinogenicity was recognized, with many scientific papers defending its use to influence policy decisions on its hazards [13]. Moreover, years after its banning, we still have not achieved an asbestos-free environment and indeed the consequences thereof we still cannot predict. 

Cancer is a multi-step process and, especially in the case of mesothelioma, it could takes years before it manifests itself. Fortunately, we are at the beginning of the CNT era and while we do not yet have data on the carcinogenicity of CNTs, we do have the opportunity to establish safe management of these materials. While we cannot precisely assess which modifications in the genome or in the epigenome will lead to mesothelioma onset, we do know that the long latency of malignant mesothelioma is sustained by decades of chronic inflammation in an aberrant microenvironment rich in ROS and the resulting oxidative DNA damage. We must carefully reflect on the data supporting the strong inflammatory potential of CNTs, similar to that of asbestos, as well as the data correlating CNT exposure with molecular alterations known to have a key role in mesothelioma onset 

## 6. Conclusions

The heterogeneity of this class of substances is undoubtedly the main obstacle to reaching a consensus on their toxicity and more studies are needed to gain detailed knowledge on the effects of exposure to CNTs. We believe that future studies on CNTs toxicity must be assessed case-by-case and, on this premise, a new evaluation of the danger of CNTs for human health is urgently needed. We strongly support the need to create a repository of biological samples from CNT-exposed workers in order to monitor biologically relevant changes over time and to encourage research collaboration within different areas of expertise. In any case, an adequate system for monitoring the health of workers exposed to this putative carcinogen remains the basis on which to build future research. 

## Figures and Tables

**Table 1:** Cancer development, histological changes, and inflammatory response observed in in vivo experiments with MWCNTs.

CNTs	Length (µm); Diameter (nm)	Cancer	Histological Changes	Inflammation	Exposure Route	Ref
Mitsui-7	L: 3–5.7 D: 49–100		x	x	intratracheal instillation	[58]
Mitsui-7	L: 3–5.7 D: 49–100	bronchiolo-alveolar adenoma and adenocarcinoma	x	x	whole body inhalation	[32]
Mitsui-7	L: 5.7 ± 0.49; D: 74 (29–173)				intratracheal instillation	[39]
Short MWCNTs	L: 1.12 ± 0.05 D: 67 ± 2		x		pharyngeal aspiration	[40]
Industrial MWCNTs	L: 2–15; D: 8–15		x	x	pharyngeal aspiration	[41]
Long MWCNTs (Nikkiso similar to Mitsui-7)	L: 1–10; D: 1–20	pleural malignant mesothelioma and lung tumors			intratracheally instilled	[59]
Long MWCNTs (University of Manchester, UK)	L: 85% > 15 D: 165 + 4.7	mesothelial hyperplasia; mesothelioma	x	x	instilled into the pleural cavity	[38]
MWCNTs (Nanostructured & Amorphous Materials, USA)	L: <15; D: 125				instilled into the pleural cavity	[38]
NM-400	L: 0.85 ± 0.10; D: 11 ± 3			x	intratracheally instilled	[39]
NM-401	L: 4.0 ± 0.37; D: 67 ± 24				intratracheal instillation	[39]
NM-402	L: 1.4 ± 0.19; D: 11 ± 3		x	x	intratracheal instillation	[58]
NM-402	L: 1.4 ± 0.19; D: 11 ± 3			x	intratracheal instillation	[39]
NM-403	L: 0.4 ± 0.03; D: 12 ± 7			x	intratracheal instillation	[39]
MWCNTs Nanotechcenter Ltd.	L: 2–15; D: 8–15		x		pharyngeal aspiration	[44]
MWCNTs(Cheaptube)	L: 0.52 (±0.59); D: 20.56 (±6.94)			x	intratracheal instillation	[60]
MWCNTs(Cheaptube)	L: 0.77 (±0.35) D: 26.73 (±6.88)			x	intratracheal instillation	[60]
MWCNTs(Cheaptube)	L: 0.72 (±1.2) D: 17.22 (±5.77)			x	intratracheal instillation	[60]

Abbreviations: “x”: studies that have reported a relationship between these characteristics and exposure to the material.

**Table 2:** Cancer development, histological changes, and inflammatory response observed in in vivo experiments with SWCNTs.

CNTs	Length (µm); Diameter (nm)	Cancer	Inflammation	Exposure Route	Ref
SWCNTs Graphistrength© C100	L: 1.06 mean; D: 11.9 mean		x	nose-only inhalation exposure	[45]
Short SWCNTs (Nikkiso & Co., LTD)	L: 0.55 ± 0.36; D: 1.4 ± 0.7	bronchiolo-alveolar adenoma and adenocarcinoma (18% of mice)		intratracheal instillation	[46]

Abbreviations: “x”: studies that have reported a relationship between these characteristics and exposure to the material.
